# The Role of Aldose Reductase in Beta-Amyloid-Induced Microglia Activation

**DOI:** 10.3390/ijms232315088

**Published:** 2022-12-01

**Authors:** Yu-Kai Huang, Chia-Chun Liu, Shining Wang, Hui-Chun Cheng, Chandler Meadows, Kun-Che Chang

**Affiliations:** 1 Graduate Institute of Medicine, College of Medicine, Kaohsiung Medical University, Kaohsiung 80708, Taiwan; 2 Division of Neurosurgery, Department of Surgery, Kaohsiung Medical University Hospital, Kaohsiung 80708, Taiwan; 3 Department of Surgery, Kaohsiung Municipal Ta-Tung Hospital, Kaohsiung 80145, Taiwan; 4 Department of Ophthalmology, Louis J. Fox Center for Vision Restoration, University of Pittsburgh School of Medicine, Pittsburgh, PA 15213, USA; 5 Department of Neurobiology, Center of Neuroscience, University of Pittsburgh School of Medicine, Pittsburgh, PA 15213, USA

**Keywords:** aldose reductase, microglia, neuron, Alzheimer’s disease, inflammation, beta-amyloid

## Abstract

The occurrence of Alzheimer’s disease has been associated with the accumulation of beta-amyloid (β-amyloid) plaques. These plaques activate microglia to secrete inflammatory molecules, which damage neurons in the brain. Thus, understanding the underlying mechanism of microglia activation can provide a therapeutic strategy for alleviating microglia-induced neuroinflammation. The aldose reductase (AR) enzyme catalyzes the reduction of glucose to sorbitol in the polyol pathway. In addition to mediating diabetic complications in hyperglycemic environments, AR also helps regulate inflammation in microglia. However, little is known about the role of AR in β-amyloid-induced inflammation in microglia and subsequent neuronal death. In this study, we confirmed that AR inhibition attenuates increased β-amyloid-induced reactive oxygen species and tumor necrosis factor α secretion by suppressing ERK signaling in BV_2_ cells. In addition, we are the first to report that AR inhibition reduced the phagocytotic capability and cell migration of BV_2_ cells in response to β-amyloid. To further investigate the protective role of the AR inhibitor sorbinil in neurons, we co-cultured β-amyloid-induced microglia with stem cell-induced neurons. sorbinil ameliorated neuronal damage in both cells in the co-culture system. In summary, our findings reveal AR regulation of microglia activation as a novel therapeutic target for Alzheimer’s disease.

## 1. Introduction

Alzheimer’s disease (AD) is one of the most common causes of memory loss and dementia, gradually causing irreversible neuronal death. In 2021, over 11 million families spent more than 16 billion hours caring for patients with AD [1]. One potential cause of AD is the formation of β-amyloid plaques in the brain, which lead to neural degeneration. Amyloidogenic processing of amyloid precursor protein (APP) causes neurotoxic β-amyloid accumulation and aggregation, which is considered to play a central role in the disease etiology [2]. The interaction of APP and β-amyloid with cell adhesion molecules subsequently induces intracellular signaling contributing to cytotoxicity [2]. In addition, the overactivation of asparaginyl endopeptidases (AEPs) cleaves tau and APP, which enhances amyloidosis and drives the onset of AD [3]. When insoluble β-amyloid accumulates in the brain, microglia infiltrate the plaque site, secreting pro-inflammatory cytokines such as tumor necrosis factor-α (TNF-α), interleukin-1β (IL-1β), interleukin-6 (IL-6), and nitric oxide [4]. The inflammatory response attacks neuronal cells around the plaques, leading to irreversible damage to the brain. In the central nervous system (CNS), microglia can play a pro-inflammatory role by secreting cytokines [5]. When microglia encounter β-amyloid in the brain, they release apoptosis-associated speck-like protein (ASC specks), activators of IL-1β and IL-18 [6], which further enhance β-amyloid aggregation. These inflammatory cytokines are mostly activated through the NF-κB and MAPK pathways [7], suggesting that suppression of these signaling pathways in microglia can inhibit β-amyloid aggregation in AD. Thus, identifying a therapeutic target to suppress pro-inflammatory signaling from microglia is a logical strategy to slow AD progression.

Aldose reductase (AR) is an enzyme that converts glucose to sorbitol in the NADPH-dependent polyol pathway of glucose metabolism [8]. AR is involved in many ocular complications such as uveitis, diabetic retinopathy [9], and posterior capsular opacification [10,11,12,13]. AR inhibition alleviates inflammatory responses in microglia, including helping reduce osmotic stress in the renal inner medulla [14]. Mouse studies show that the deletion of AR ameliorates hyperglycemia-induced ocular complications [15,16]. In contrast, increased AR in transgenic mice increases microglia migration into the inner and outer nuclear layers of the retina [17]. Another study showed that activated microglia infiltrated into the retina of transgenic mice overexpressing AR, resulting in retinal ganglion cell (RGC) loss [18]. Because AR plays a critical role in microglia activation and retinal neurodegeneration, we asked whether AR also regulates β-amyloid-induced microglial activation.

Pathogen invasion or trauma in the CNS triggers microglia migration to the lesion site. There, surveillance microglia attack pathogens by secreting cytokines or cleaning up dying neurons by phagocytosis [19]. However, excessive cytokine secretion and phagocytosis may stress healthy neurons. Thus, it is critical to understand what regulates microglia activation. ERK, one of the MAPK family proteins, is an upstream regulator of cell migration [20] and pro-inflammatory cytokine production [21]. Our previous study showed that AR inhibition reduces lipopolysaccharide-induced ERK activation in macrophages [22]. However, it is not known whether AR inhibition can also suppress ERK and its downstream signaling in β-amyloid-treated microglia. To examine whether AR is a viable target for AD treatment, we first validated the effects of an AR inhibitor, sorbinil, on β-amyloid-induced pro-inflammatory cytokine secretion in microglia. We next demonstrated that sorbinil treatment alleviates β-amyloid-induced reactive oxygen species (ROS) and ERK activation in microglia. We report for the first time that AR inhibition prevented cell migration and phagocytosis in β-amyloid-treated microglia. Finally, we showed that AR inhibition in microglia attenuated neuronal death in a microglia–neuron co-culture system. The results of our studies substantiate the case for sorbinil as a potent and effective therapeutic agent against β-amyloid-induced neural degeneration and, thus, a potential therapy for AD.

## 2. Results

### 2.1. AR Inhibition Alleviates β-Amyloid-Induced TNF-α Secretion in Microglia

**Figure 1:**
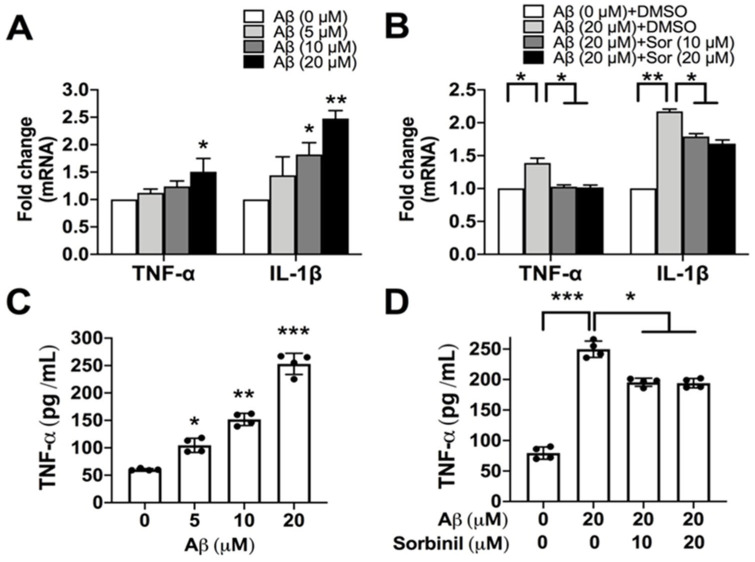
Aldose reductase (AR) inhibition attenuates inflammatory cytokine expression in BV_2_ cells. (**A**) qRT-PCR results showing beta-amyloid (Aβ)-induced expression of tumor necrosis factor α (*TNF-α*) and *IL-1β* mRNA in a dose-dependent manner in BV_2_ cells. (**B**) Effect of treatment with DMSO vehicle or sorbinil on *TNF-α* and *IL-1β* mRNA expression. (**C**) Quantification of TNF-α protein expression from Western blots of BV_2_ cells treated with Aβ. (**D**) Effect of treatment with sorbinil on TNF-α protein expression. Data shown are means ± SEM (N = 3). * *p* < 0.05, ** *p* < 0.01, *** *p* < 0.005.

β-amyloid precipitation in the brain induces inflammation and gradually causes neurodegeneration, one of the leading causes of AD. To understand whether AR is involved in β-amyloid-induced inflammation, we first added β-amyloid to microglia cultures. β-amyloid induced *TNF-α* and *IL-1β* mRNA expression in BV_2_ cells in a dose-dependent manner (Figure 1A). We next applied sorbinil, a well-known AR inhibitor used for neural disorders in a rodent model [23], to the cultures and found that sorbinil significantly reduced both *TNF-α* and *IL-1β* expression (Figure 1B). In addition to gene expression, we also investigated the protein expression of TNF-α and observed similar trends (Figure 1C,D), suggesting that sorbinil can attenuate β-amyloid-induced inflammatory responses in BV_2_ cells. Because 10 μM of sorbinil showed similar effects to 20 μM, we used 10 μM of sorbinil for subsequent studies.

### 2.2. AR Inhibition Decreases β-Amyloid-Induced ROS Production

**Figure 2:**
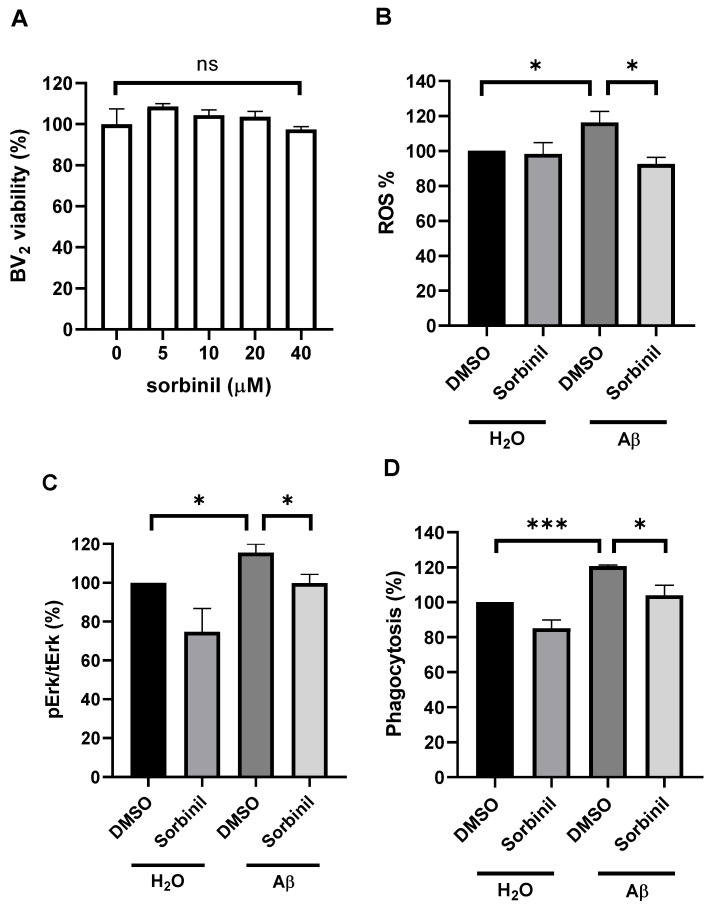
AR inhibition reduces reactive oxygen species (ROS) production and ERK signaling in BV_2_ cells. (**A**) Results of MTT cell viability assay of BV_2_ cells treated with a range of sorbinil doses. (**B**) ROS assay results for BV_2_ cells pretreated with sorbinil (10 µM, 1 h) and incubated with β-amyloid (Aβ; 10 µM, 1 h). (**C**) Phospho-ERK production in BV_2_ cells pretreated with sorbinil (10 µM, 1 h) and treated with Aβ (10 µM, 24 h) was measured by Western blot. (**D**) Phagocytosis assay results for BV_2_ cells pretreated with sorbinil (10 µM, 1 h) and treated with Aβ (10 µM, 1 h). DMSO and H_2_O represent negative controls. Data shown are means ± SEM (N = 5). * *p* < 0.05, *** *p* < 0.005, ns = not significant.

To test whether sorbinil is toxic to microglia, BV_2_ cells were treated with various dosages of sorbinil. We observed no significant change in cell viability after treatment with up to 40 μM sorbinil (Figure 2A), suggesting that an effective dosage of sorbinil is non-toxic in cultured microglia. In microglia, activation of MAPK signaling produces ROS [11], and AR is a key regulator of these signaling pathways [24]. Because increased ROS is involved in inflammatory cytokine secretion [25] and the NF-κB pathway [26], we next asked whether the AR polyol pathway regulates β-amyloid-induced ROS production [27]. β-amyloid induced ROS in BV_2_ cells, and this effect was suppressed by sorbinil treatment (Figure 2B), suggesting that AR inhibition can reduce oxidative stress in microglia. A previous study showed that ROS induce ERK signaling [28]. Our data further demonstrated that AR inhibition reduces the phosphorylation of ERK (Figure 2C), which is a key regulatory signal for inflammatory cytokine production [29]. Microglia perform phagocytosis to remove pathogens or apoptotic cells [30]. To investigate whether AR regulates phagocytosis, we pretreated BV_2_ cells with sorbinil following β-amyloid treatment and found that sorbinil treatment decreased phagocytosis by 27% (Figure 2D).

### 2.3. AR Inhibition Attenuates β-Amyloid-Induced BV_2_ Cell Migration

**Figure 3:**
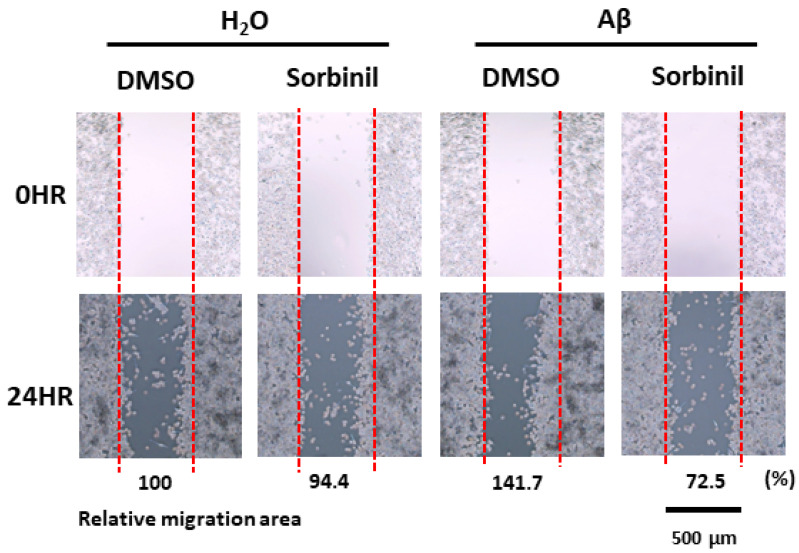
AR inhibition decelerates cell migration in BV_2_ cells. Representative images of migration of BV_2_ cells after treatment with β-amyloid (Aβ; 10 µM) or H_2_O control and treatment with sorbinil (10 µM) or DMSO control. Numbers at the bottom of images show relative migration area of BV_2_ cells in each group compared to untreated controls.

CNS injury or infection activates and recruits surveillance microglia to the injury site. In AD, activated microglia migrate to amyloid plaques [31] and may accelerate tissue damage by secreting more inflammatory molecules. Because AR regulates microglia migration after endotoxin exposure [32,33], we asked whether AR plays a similar role in response to β-amyloid. By co-treating BV_2_ cells with β-amyloid and sorbinil for 1 day, we found that β-amyloid promotes BV_2_ cell migration, which is attenuated by sorbinil treatment (Figure 3). This result indicates that AR inhibition alleviates activated microglia migration.

### 2.4. AR Inhibition in BV_2_ Cells Protects Neurons from Death under β-Amyloid Exposure

**Figure 4:**
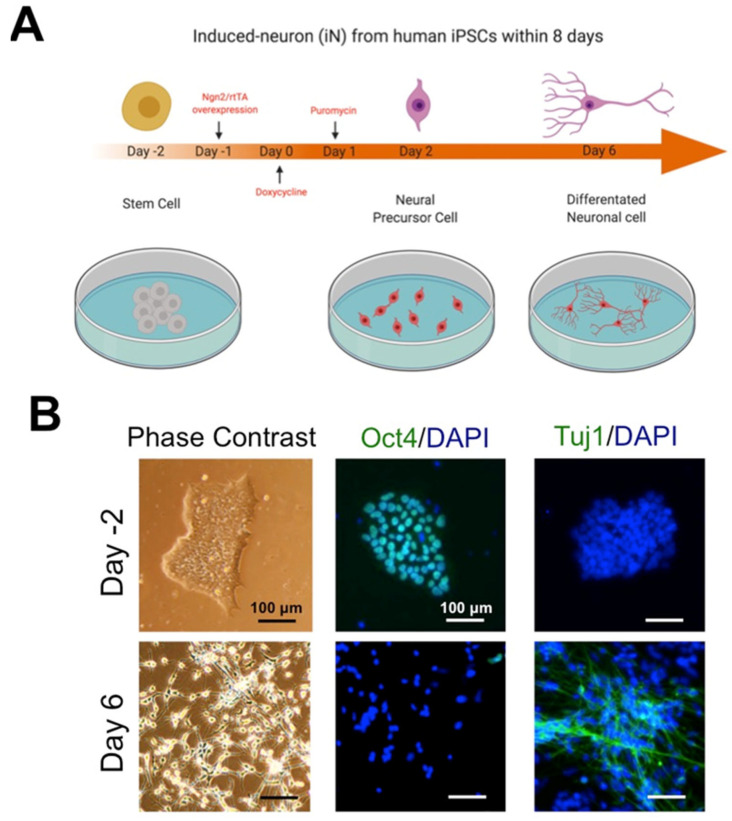
Induced neuron (iN) differentiation from human stem cells. (**A**) Schematic for differentiation of human induced pluripotent stem cells (hiPSCs) into retinal ganglion-like cells. (**B**) Staining for Oct4 and neural marker Tuj1, representing stem cell pluripotency and neurite formation, respectively, in iNs. Scale bar = 100 μm.

We next asked whether sorbinil can protect human neuronal cells from β-amyloid-induced damage. We differentiated hiPSCs to iNs by overexpressing Ngn2 (Figure 4A), following a previously described protocol with minor modification [34]. On day 2, pluripotent stem cell marker Oct4, but not neuronal marker Tuj1, was observed in the culture (Figure 4B). After cell differentiation for 8 days, we observed Tuj1 expression but no longer observed Oct 4 expression (Figure 4B).

**Figure 5:**
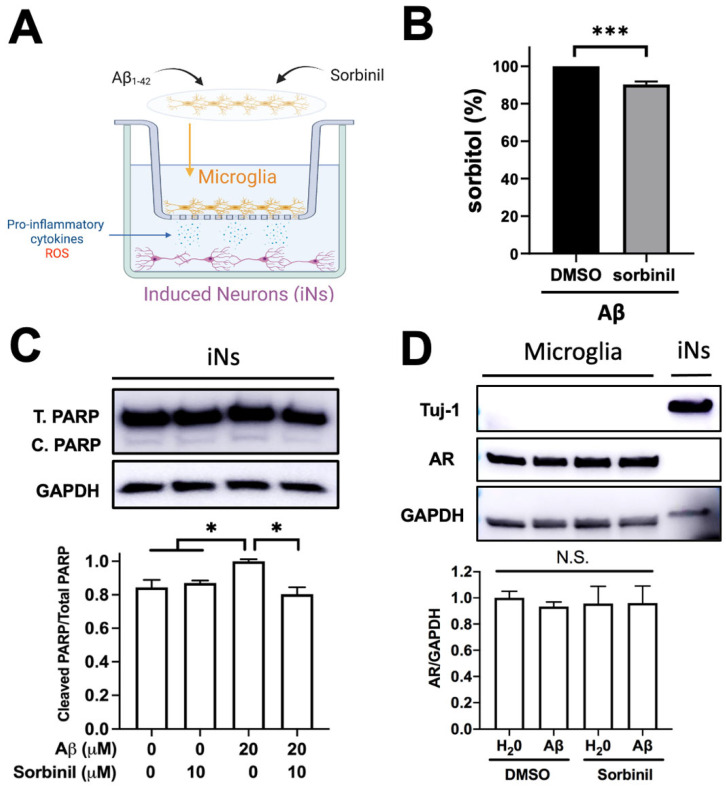
AR inhibition alleviates neuron death in microglia–induced neuron (iN) co-culture. (**A**) Diagram of co-culture setup for microglia and iNs. (**B**) Sorbitol assay indicated that sorbinil would attenuate sorbitol accumulation in the BV_2_ cells. (**C**) Western blots and corresponding ratio of cleaved-PARP (C. PARP) over total-PARP (T. PARP) expression in iNs following treatment with β-amyloid (Aβ) and/or sorbinil. (**D**) Western blots and corresponding quantification of AR and Tuj1 protein expression in microglia and iNs following treatment with Aβ and/or sorbinil. GAPDH is a loading control. Data shown are means ± SEM (N ≥ 3). * *p* < 0.05, *** *p* < 0.005, N.S. = not significant.

To mimic the in vivo microenvironment, we first treated BV_2_ cells with β-amyloid and sorbinil and then co-cultured the activated BV_2_ cells with iNs in the present sorbinil (Figure 5A). In addition, we confirmed that sorbinil inhibits the AR polyol pathway in BV_2_ cells by a sorbitol accumulation study (Figure 5B), even though β-amyloid didn’t increase sorbitol accumulation. Our previous data showed that β-amyloid activates microglia, which results in the secretion of pro-inflammatory cytokines into the medium (Figure 1). To assess apoptotic effects, we investigated cleaved PARP, which is involved in DNA repair during environmental stress [35]. Because 10 μM β-amyloid did not cause any cell death in iNs, we increased the dose to 20 μM and observed an increase in cleaved PARP in the presence of β-amyloid, which was attenuated by sorbinil treatment (Figure 5C). Further, no AR was expressed in iNs, and neither β-amyloid nor sorbinil altered AR protein expression (Figure 5D), suggesting that a limited off-target effect of sorbinil would occur on iNs and sorbinil mainly inhibits the enzymatic feature of AR, not its expression. These data suggest that AR inhibition could alleviate inflammatory responses and prevent neuron death in the brain in the context of β-amyloid plaque accumulation.

## 3. Discussion

**Figure 6:**
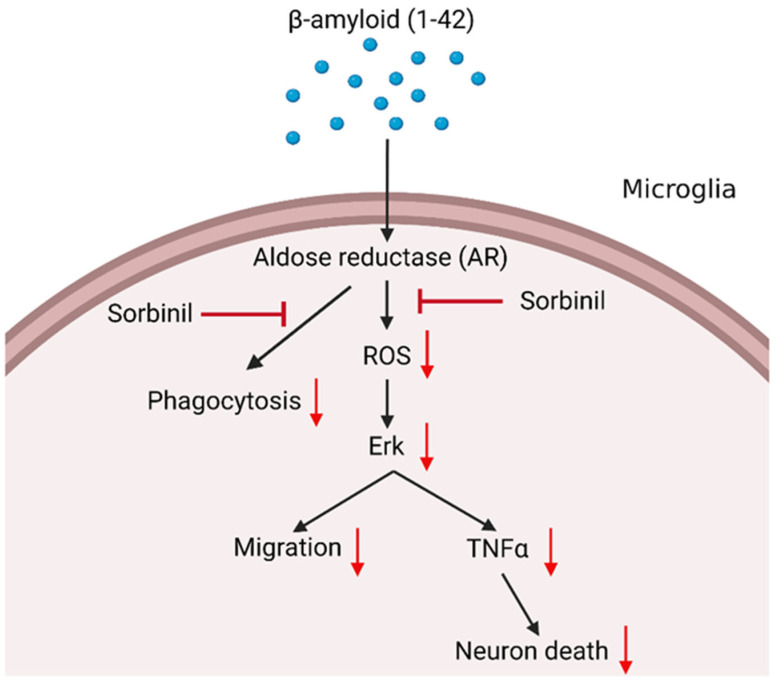
Proposed role of AR in microglia and subsequent neuron death caused by β-amyloid exposure. β-amyloid induces the production of reactive oxygen species (ROS), TNF-⍺ secretion, cell migration, and phagocytosis in microglia. We propose that AR inhibition attenuates the inflammatory responses and prevents neuronal death caused by increased inflammatory cytokine secretion. Red arrows indicate attenuation.

In summary, β-amyloid application (or accumulation) activates surveillance microglia, leading to ROS production, elevated TNF-α secretion, and increased phagocytosis, which results in neuron death [36] (Figure 6). The increase in microglial migration ability might cause a larger area of brain damage. AR inhibition prevents microglia activation and migration, which could alleviate neuron death and slow the progression of AD.

In the condition of β-amyloid accumulation, surrounding microglia become activated and play a critical role in inflammation and its subsequent pathogenesis [6]. Thus, understanding what regulates microglia activation is important to alleviate inflammatory responses. The AR polyol pathway induces inflammation by activating ROS production as well as NF-κB and p38/MAPK signaling [22,24,37,38,39,40]. In the eyes, AR inhibition alleviates endotoxin-induced microglia activation in the retina [17,32,41] and AR overexpression leads to RGC loss [18]. We next ask whether results taken from RGCs can be extrapolated to the rest of the brain/CNS. Several studies show that the blockade of AR protects the CNS by suppressing microglia activation [38,42,43]. A recent study demonstrated that microglia play a significant role in the infiltration of β-amyloid pathology into healthy parts of brain tissue [44]. Although AR had been previously reported to be involved in β-amyloid-induced microglia activation [24], whether AR inhibition could reduce microglia migration and prevent neuronal death remained unknown.

In in vitro studies, we found that pro-inflammatory cytokines were induced by β-amyloid exposure and attenuated by sorbinil treatment, which is consistent with our and others’ previous findings that AR inhibition can suppress microglia activation [17,24,32,45]. We further showed that sorbinil treatment can reduce β-amyloid-induced ROS production and pERK activation in BV_2_ cells, providing an underlying mechanism of AR in microglia activation by β-amyloid exposure. Sorbitol is the metabolic product of the AR polyol pathway [9]. Our data show that sorbinil can decelerate sorbitol accumulation in microglia, suggesting that sorbinil regulates microglia physiology in an enzymatic and AR-dependent manner. In our co-culture study, we did not see the AR expression and sorbitol accumulation change after β-amyloid treatment, indicating that β-amyloid may not increase the AR polyol pathway. However, AR inhibition is known to suppress inflammatory signaling via an alternative mechanism. For example, the AR polyol pathway leads to the decrease of NAD+/NADH ratio, which upregulates Sirt1-mediated inflammation and apoptosis [9,46,47] and promotes ROS production. Inhibition of the AR polyol pathway thus suppresses inflammatory response in the cell, providing another regulatory mechanism of AR in microglial activation. Previous research indicates that the reduction of phagocytosis in microglia promotes healthy neuron survival [48]. In addition to removing dead cells or cellular debris, phagocytosis is also critical to some neurodevelopmental diseases [49]. Thus, studying the role of AR in neurodevelopmental disorders would be an interesting direction in the future. As microglia can switch from M1 to M2 to play a protective role [5], it would be interesting to know whether sorbinil affects the M1/M2 regulation of microglia. This could also be an interesting area of investigation in a future study.

Sorbinil treatment reduced the migration ability of β-amyloid-activated microglia, which suggests its potential to protect unaffected areas of the brain from damage. Our previous studies showed that the blockade of AR inhibits matrix metalloproteinase (MMP)-9-medicated macrophage migration [32]. Because ROS controls the MMP-9 cascade [50], and macrophages and microglia are physiologically similar regarding neural inflammation, this suggests that the mechanism of β-amyloid-activated BV_2_ cell migration is likely the same in this case, regulated by the MMP-9 cascade.

Due to the limited resource of human neurons, in vitro study of AD has been a challenge. Human stem cell-derived neurons provide the possibility of using human neurons for AD study. The ability to differentiate iNs via Ngn2 overexpression significantly benefits the study of AD [34,51,52,53]. In this study, iNs were used to determine the protective role of AR inhibition in neuronal death caused by β-amyloid-activated microglia. The co-culture system we used here models a similar environment to the human brain and can be used to screen potential molecules for AD treatment. We observed that sorbinil prevents neurons from activated BV_2_ cell-induced apoptosis without changing AR protein expression, suggesting that sorbinil inhibits the AR polyol pathway but not protein expression. In addition, we did not observe AR in iNs and any neuronal cytotoxicity of sorbinil on iNs in the co-culture culture, indicating the specificity of sorbinil in microglia. These data warrant that AR is a potential therapeutic target for microglia-relevant neurodegenerative disorders and suggest that applying sorbinil to the AD animal model would be an excellent future direction. Although the iNs model facilitates our study, there is still a limitation in that 2D stem cell-derived neurons do not represent the real cell–cell interaction in the brain. Cerebral organoids could be a better model for such a study in the future. In addition, hyperphosphorylation of Tau protein causes neurofibrillary tangles in AD and related tauopathies [54,55]. Since AR inhibition suppresses MAPK protein phosphorylation [22], whether AR also regulates tauopathies remains unknown and is a topic for future work.

A variety of AR inhibitors (ARIs) have been developed, although liver and/or renal toxicity remains a concern [56,57,58]. Thus, developing an effective but low-cytotoxic ARI remains a goal for the treatment of microglia-associated inflammation [59,60]. Fruits such as Indian gooseberry contain a natural ARI called beta-glucogallin, which has relatively low cytotoxicity to murine macrophages and can prevent lipopolysaccharide-induced inflammatory cellular infiltrates in the mouse eye [22] via reduced sorbitol accumulation in macrophages. The reduction of sorbitol diminishes osmotic stress and oxidative stress [61], contributing to cell survival. Many natural ARIs have been identified in fruits [62], and it would be an interesting area of future study to investigate whether ARIs play a protective role in neurodegenerative diseases, as daily consumption of fruits that contain natural ARIs could be an adjuvant for delaying the onset or progression of AD.

In summary, we reported the beneficial role of ARI in neurodegenerative disease by suppressing inflammatory responses using a neuron-microglia co-culture system. This study would be insightful to the field of pharmaceutical sciences for ARI development as the therapeutic agent to reduce inflammatory responses and phagocytosis in microglia cells and to prevent surrounding neuronal cell death in neurodegenerative diseases such as AD.

## 4. Materials and Methods

### 4.1. Cell Culture

Mouse BV_2_ cells were obtained from ATCC (Manassas, VA, USA) and cultured in DMEM/F12 medium (Invitrogen, Waltham, MA, USA) containing 5% penicillin/streptomycin and 10% fetal bovine serum. Human induced pluripotent stem cells (hiPSCs) were derived from peripheral blood mononuclear cells of a healthy male donor, and the cell line was obtained from Stanford Stem Cell Core. hiPSCs were treated with EDTA (Sigma-Aldrich, St. Louis, MO, USA) and plated as dissociated cells in 24-well plates (hiPSCs: 1.5 × 10^4^ cells/well). Neurons were differentiated from iPSCs as previously described [34]. Briefly, cells were plated on Matrigel (Corning Inc., Corning, NY, USA)-coated plates in mTeSR™1 (Stemcell Technologies, Vancouver, BC, Canada) containing 2 μM thiazovivin (BioVision, Miltas, CA, USA). Lentivirus including neurognin2 (Ngn2), rtTA, and eGFP (0.3 μL/well of 24-well plate) were added in fresh mTeSR™1 medium containing polybrene (8 μg/μL, Sigma-Aldrich). On day 0, culture medium was replaced with N2/DMEM/F12/NEAA (Invitrogen) containing human BDNF (10 μg/L, PeproTech, Cranbury, NJ, USA), human NT-3 (10 μg/L, PeproTech). Doxycycline (2 mg/mL, Sigma-Aldrich) was added on day 0 to induce TetO gene expression and was retained in the medium until the end of the experiment. On day 1, a 24 h puromycin selection (2 mg/mL) period was started. On day 2, GDNF (50 ng/mL) was added in Neurobasal medium supplemented with B27/Glutamax (Invitrogen) and containing BDNF and NT3. After day 2, half of the medium in each well was exchanged every 2 days. Induced neurons (iNs) were assayed on day 8 in most experiments.

### 4.2. Aβ 1-42 Preparation

β -amyloid peptide 1-42 were purchased from Sigma-Aldrich (AG912), prepared as described previously [63], after dissolving β-amyloid with the ddH_2_O at a concentration of 100 μM, then incubated at the 37 °C for 1 week. The fibrils were observed after incubation.

### 4.3. Co-Culture Assay

iNs (1.5 × 10^4^ cells/well) were placed in the bottom of a 24-well plate. BV_2_ cells were coated at a density of 3 × 10^5^ cells/mL in a 6.5-mm transwell dish (Corning) pretreated with sorbinil (10 µM) for 1 h and then treated with β-amyloid (10 µM) for another 6 h before co-culture. Activated BV_2_ cells were then moved to the wells of iNs for co-culture in neuron differentiation medium with sorbinil for 24 h before neuronal lysate collection.

### 4.4. ROS Assay

Established BV_2_ cells were coated at a density of 1 × 10^5^ cells/well in a 96-well plate. BV_2_ cells were pretreated with β-amyloid and sorbinil (10 µM) for 1 h. The medium was removed and 50 µL H2DCFA (5 µM, #C6827, Invitrogen) dissolved in DMSO and DPBS was added for 30 min at 37 °C. Cells were washed with DPBS and incubated with 10 µM β-amyloid peptide 1–42 (Aβ_1-42_) and sorbinil (10 µM) for 1 h. Medium was removed and replaced with PBS to not interfere with the results. The assay was read at excitation/emission wavelengths of 488 nm/525 nm.

### 4.5. ELISA Assay

BV_2_ cells (10^5^ cells/well) were incubated in a 24-well plate, and media were collected after β-amyloid treatment. Secreted TNF-α in media was measured using a mouse TNF-α DuoSet ELISA Development kit (R&D Systems Inc., Minneapolis, MN, USA) and mouse IL-1β DuoSet kit (R&D Systems Inc.). Optical density was detected using a Synergy 4 Hybrid microplate reader (BioTek, Winooski, VT, USA), and cytokine level was deducted from the absorbance value by extrapolation from a standard curve generated in parallel.

### 4.6. Real-Time qPCR (RT-qPCR)

BV_2_ cells (5 × 10^5^ cells/well) were incubated in a 12-well plate, and cells were collected after β-amyloid treatment for RNA extraction. mRNA from cells were extracted by TRIzol (Invitrogen, Carlsbad, CA, USA) and converted into cDNA using the Applied Biosystems kit (Applied Biosystems, Waltham, MA, USA). RT-PCR for gene expression was performed with specific primer sets by TaqMan gene expression master mix (#4369016) in Applied Biosystems StepOnePlus qPCR system. Primers for qPCR are as follows: mouse TNF-α: forward 5′-ATGAGCACAGAAAGCATGATCCGC-3′, reverse 5′-CCAAAGTAGACCTGCCCGGACTC-3′; mouse IL-1β: forward 5′-TCAGGCAGGCAGTATCACTCA-3′, reverse 5′-GGAAGGTC-CACGGGAAAGAC-3′.

### 4.7. Western Blot

After co-culture, iNs were collected and washed with PBS. RIPA buffer with phosphatase inhibitor was added, and cells were sonicated for 10 s. After centrifugation at 13,500 rpm for 5 min, proteins contained in the supernatant were determined by Thermo Scientific BCA protein assay. Equal amounts of protein lysates (50 µg) were separated by SDS-PAGE and transferred to a PVDF membrane (Bio-Rad, Hercules, CA, USA) using a semi-dry blotter (Bio-Rad). Membranes were blocked with blocking buffer (LI-COR) and probed with primary antibodies p-ERK, ERK, AR, Tuj-1, cleaved poly (ADP-ribose) polymerase (PARP), vinculin, and GAPDH (1:1000, Cell Signaling Technology, Danvers, MA, USA) in antibody diluent buffer overnight at 4 °C. Membranes were washed with TBST buffer and probed with fluorescence-conjugated secondary antibodies, imaged by the Odyssey M Imaging System (LI-COR Biosciences, Lincoln, NE, USA).

### 4.8. Migration Assay

BV_2_ cells were seeded at a density of 3 × 10^5^ cells/mL in a Culture-Insert 2 Well dish (81176, Ibidi, Gräfelfing, Germany) containing 70 μL of medium/well and incubated overnight. BV_2_ cells were pretreated with or without sorbinil (10 µM) for 1 h. After gently removing the barrier well, medium was removed. β-amyloid and sorbinil were added for an additional 24 h, and cell migration was observed by EVOS XL Core microscope (Invitrogen). Quantification was performed by measuring the area into which cells migrated.

### 4.9. Phagocytosis Assay

BV_2_ cells were seeded at a density of 5 × 10^5^ cells/mL in a 96-well plate. BV_2_ cells were pretreated with sorbinil (10 µM) for 1 h. Medium was removed, and β-amyloid peptide 1-42 (Aβ_1-42_) (10 µM) was added as the phagocytosis effector with sorbinil (10 µM) for 1 h. Medium was again removed, and bioparticles from a Vybrant kit (V-6694, Molecular Probes, Eugene, OR, USA) were added for 2 h. An equal volume of trypan blue was used to quench the extracellular probe. Trypan blue was removed, and the assay was read with excitation/emission wavelengths of 480 nm/520 nm.

### 4.10. Statistical Analysis

Results are shown as mean ± SEM of at least three experiments. Data were analyzed by ANOVA with post hoc *t*-tests or Student’s *t*-test with *p* < 0.05 considered significant.

## Data Availability

The data that support the findings of this study are available from the corresponding author (KCC) upon request.
